# Encodings and models for antimicrobial peptide classification for multi-resistant pathogens

**DOI:** 10.1186/s13040-019-0196-x

**Published:** 2019-03-04

**Authors:** Sebastian Spänig, Dominik Heider

**Affiliations:** Department of Bioinformatics, Faculty of Mathematics and Computer Science, Philipps-University of Marburg, Marburg, Germany

**Keywords:** Machine learning, Antimicrobial peptides, Encodings

## Abstract

Antimicrobial peptides (AMPs) are part of the inherent immune system. In fact, they occur in almost all organisms including, e.g., plants, animals, and humans. Remarkably, they show effectivity also against multi-resistant pathogens with a high selectivity. This is especially crucial in times, where society is faced with the major threat of an ever-increasing amount of antibiotic resistant microbes. In addition, AMPs can also exhibit antitumor and antiviral effects, thus a variety of scientific studies dealt with the prediction of active peptides in recent years. Due to their potential, even the pharmaceutical industry is keen on discovering and developing novel AMPs. However, AMPs are difficult to verify in vitro, hence researchers conduct sequence similarity experiments against known, active peptides. Unfortunately, this approach is very time-consuming and limits potential candidates to sequences with a high similarity to known AMPs. Machine learning methods offer the opportunity to explore the huge space of sequence variations in a timely manner. These algorithms have, in principal, paved the way for an automated discovery of AMPs. However, machine learning models require a numerical input, thus an informative encoding is very important. Unfortunately, developing an appropriate encoding is a major challenge, which has not been entirely solved so far. For this reason, the development of novel amino acid encodings is established as a stand-alone research branch. The present review introduces state-of-the-art encodings of amino acids as well as their properties in sequence and structure based aggregation. Moreover, albeit a well-chosen encoding is essential, performant classifiers are required, which is reflected by a tendency towards specifically designed models in the literature. Furthermore, we introduce these models with a particular focus on encodings derived from support vector machines and deep learning approaches. Albeit a strong focus has been set on AMP predictions, not all of the mentioned encodings have been elaborated as part of antimicrobial research studies, but rather as general protein or peptide representations.

## Introduction

Antimicrobial peptides are part of the inherent immune system of almost all organisms, such as plants, animals, and humans [1]. Owing to increasing rates of multi-resistant pathogens, the scientific community has reached out for novel strategies to tackle this threat [2, 3]. One of these approaches leverages the endogenous defense system mode of action, particularly on exposed surfaces, such as the skin, commonly referred to as antimicrobial peptides (AMPs) [1]. To this end, researchers have shown that AMPs also have an effect even against multi-resistant pathogens and thus, can effectively employed as antibiotic agents. AMPs can also interfere intracellular mechanisms, which makes these potential candidates for cancer treatment or inflammatory diseases [4]. Owing to their broad fields of application and the demonstrated potential, the pharmaceutical industry pushes research ahead in order to discover and develop novel and highly effective AMPs, such as the approved polymyxins, which serve as last resort therapy, if the usual treatment fails [4]. In order to enable AMP detection with low costs and in high throughput, computational approaches offer the opportunity to explore the huge space of sequence variations in a timely manner. In particular, artificial intelligence, hence machine learning algorithms perform well in prediction and classification tasks, including computer vision [5], autonomous driving [6], or life science [7]. It is thus not surprising, that machine learning has been applied for fast and automated discovery of AMPs [8] and protein classification in general [9]. Two major issues arise here: firstly, biological information of the amino acid sequence has to be translated into a numerical representation and secondly, the input must not be of varying length, therefore sequence lengths have to be aligned. This is due to the intrinsic nature of machine learning models, i.e., the requirement of a numerical input with a fixed dimension. To this end, a variety of encodings has been developed over time. Each of these encodings are created to reflect biological relationships as well as intrinsic information of the primary sequence and higher order confirmations as accurate as possible. Since an informative encoding is very important and crucial for prediction accuracy, not only numerous encodings have been proposed, but also various strategies to combine existing ones. In order to shed light in this complex topic, literature has been mined for sequence and structure based encodings and elaborated as part of this review. The goal of the present study is the easing of the application of existing encodings for own projects and to encourage further research in the automated classification of antimicrobial peptides. The paper is structured as follows: in order to understand the rationale behind different encodings, we introduce the general effect of AMPs in the first section. Afterwards, prepared with the biological background, we summarize sequence- and subsequently structure-based encodings in the second section. Since the prediction task requires not only an expressive encoding, but also a performant classifier, we further highlight the employed machine learning algorithms in another section. Moreover, special encodings have been derived from support vector machines and deep learning. For this reason, we elaborate on these more detailed in another section. For the sake of completeness, tools for AMP prediction are uncovered, which includes different databases as sources for AMP sequences and packages, which provide implementations for many of the presented encodings.

## Antimicrobial peptides

AMPs are part of the inherent immune system and can be especially found in exposed surfaces, such as mucosa and the skin [1]. At these sites, AMPs serve as a defence system and are expressed to protect the organism against microbial intruders. The defense measures encompasses different types of bacterial interaction, mostly due to the AMPs physicochemical properties and the resulting three-dimensional structure. That is, mostly positive charged and hydrophobic residues are constituted to 10 to 50 residues long peptides, forming either α-helices, β-sheets or random coils [1]. Due to the “multi-hit mechanism”, adaption against AMPs is difficult and thus, AMPs are effective even against highly resistant pathogens. To this end, active peptides are interacting with pathogens in two ways: on the one hand, they disrupt the bacterial membrane and on the other hand, they advance further into the cell, generally known as translocation [10]. Because of different characteristics of eukaryotic and prokaryotic membranes, the interaction of AMPs with their corresponding target is highly selective [11]. The membrane disruption leads to the loss of important ions and metabolites, which finally leads to cell lysis and subsequently to cell death [1]. Essentially, three membrane disruption models are known: the barrel-stave model for pore building, the carpet model for disintegration of the membrane, as well as the toroidal-pore model for arranging the membrane to build continuous pores [1, 11]. The further advancement to intracellular location, i.e., translocation, takes place without permeabilizing the pathogens membrane. Within the cell, AMPs aggregate in the cytoplasm and inhibit nucleic acid as well as protein synthesis [12]. Besides antimicrobial effects, antiparasitic, antivirus, and anticancer effects have been reported. In the case of the latter, AMPs can trigger apoptosis and prevent angiogenesis [4].

While most AMPs have the ability to kill microbial pathogens directly, other peptides, e.g., anticancer AMPs, have immunomodulatory capabilities to stimulate cells and tissues of the host defense system. More general, these class of peptides are known as host defense peptides (HDP). For instance, the well-studied HDP LL-37 [13] reveals its complex mode of action, due to direct and indirect interactions with a vast amount of genes and proteins of the host. Hence, HDPs are important signaling molecules, capable, for instance, to regulate autoimmune response in the case of inflammatory diseases or, as mentioned above, support tumor suppression [14].

## Encodings

This section describes the different approaches and mechanisms to encode an amino acid sequence as a numerical vector and is divided in two main parts: the first deals with sequence-based encodings and the second part describes structure-based encodings. The former, summarized in Table 1, encompass sparse or binary encoding, followed by the general and the pseudo-amino acid composition. Afterwards, the reduced amino acid alphabet will be introduced as well as descriptors, which incorporate physicochemical as well as statistical properties of the respective amino acid and substitution matrices (which incorporate the substitution frequency of amino acids). Nevertheless, the function of a peptide is defined by its three-dimensional shape, hence structure-based encodings (Table 2) have been proposed in order to improve prediction performances. Thus the second part of this section introduces structure-based encodings. Besides the classical state-of-the-art approaches for encoding of peptides, novel, promising encodings have been developed, such as the Chaos Game Representation, which are described in the third section and summarized in Table 3. Hereinafter, each of these encodings are compared in detail and applications and method specific customizations are provided as well as, if possible, the relation between the biology behind the encodings and the antimicrobial effect.

**Table 1:** Summary of sequence based encodings

Encoding	Description	Summary	Used in	Used along with	Main Category
Sparse	each amino acid is represented as an one-hot vector of length 20, where each position, except one, is set to 0	Density: - Information: +	[15, 19–21]	Substitution Matrix, Amino Acid Composition	Sparse encoding
Amino Acid Composition	feature vector contains at each position the proportion of an amino acid in relation with the sequence length	Density: + Information: -	[22–24]	Distance Frequency, Quantitative Matrix, Dipeptide Composition, PseAAC	Amino acid composition
Distance Frequency	calculates the distance between amino acids of similar properties and bins the occurrence according to the gap length	Density: + Information: +	[22]		Amino acid composition
Quantitative Matrix	encodes the propensity of each amino acid at a position	Density: + Information: +	[23]		Amino acid composition
CTD	describes the composition (C), transition (T) and distribution (D) of similar amino acids along the peptide sequence	Density: + Information: +	[25]		Amino acid composition
Pseudo-amino Acid Composition (PseAAC)	computes the correlation between different ranges among a pair of amino acids	Density: + Information: +	[27–30]	Dipeptide Composition	Pseudo amino acid composition
Reduced Amino Acid Alphabet	similar amino acids are grouped together	Density: + Information: o	[9, 32–34, 36, 37]	N-gram Model, AAIndexLoc	Reduced amino acid alphabet
N-gram Model	occurrences of n-mers for an alphabet of size m, leading to a m^n^ dimensional, sparse representation of the initial sequence	Density: - Information: o	[9]		Reduced amino acid alphabet
AAIndexLoc	k-nearest neighbor clustering to aggregate amino acids into 5 classes using their amino acid index, i.e., amino acids with the respective highest(T), high (H), medium (M), low (L), and lowest (B) values of a particular physicochemical property are clustered together	Density: o Information: +	[37]	Dipeptide Composition	Reduced amino acid alphabet
Physicochemical Properties	translation of an amino acid to a particular physicochemical property	Density: o Information: +	[40, 42, 47–53]	z-descriptor, d-descriptor and many more	Physicochemical properties
z-descriptor	derived from the principal components of physicochemical properties by means of partial least squares (PLS) projections, PLS leads to a subset of five final features, capable to describe the 20 proteinogenic as well as 67 additional amino acids	Density: + Information: +	[42, 44]		Physicochemical properties
d-descriptor	amino acid sequence is squeezed between the y- (N-terminus) and the x-axis (C-terminus) with gradually bending of the single amino acids and subsequent vector summation	Density: + Information: +	[54]		Physicochemical properties
Autocorrelation	interdependence between two distant amino acids in a peptide sequence	Density: + Information: +	[57–61]		Autocorrelation
Substitution/Scoring Matrix	provide accepted mutations between amino acid pairs, i.e., sequence alterations with either no or positive impact in terms of the protein function	Density: + Information: +	[65–71]	BLOMAP, Sparse, Amino Acid Composition, Dipeptide Composition, PseAAC, AAIndexLoc	Substitution and scoring matrix
BLOMAP	incorporates the BLOSUM62 to calculate distances in a high dimensional input space, i.e., the substitution matrix, to a lower dimension, using the Shannon-projection	Density: + Information: +	[65]		Substitution and scoring matrix
Fourier Transformation	to detect underlying patterns in time series, by transforming the time signal to a frequency domain	Density: o Information: +	[73, 74]		Fourier Transformation

+ (good), o (neutral/no declaration), − (bad). For instance, “Density: -” means the encoding results in a high dimensional feature space and “Information: +” reflects a representative mapping from the residue sequence to the numerical vector. “o” denotes encodings, which are difficult to classify, due to missing details in the respective publication or can be considered as neutral. In general, the classification rests upon the authors experience and shall support researchers to quickly grasp suitable encodings. Nevertheless, an encoding which has been rated “-” still might work well for a particular application and should by no means regarded as the final evaluation

**Table 2:** Summary of structure derived encodings

Encoding	Description	Summary	Used in	Used along with
Quantitative structure-activity relationship (QSAR)	describes amino acids sequences by their chemical properties, molecular characteristics and structure	Density: o Information: +	[78–85]	z-Descriptors
General Structure	protein structure is described by means of their total 3D shape, secondary structure, solvent accessibility, aggregation tendency, contact number, residue depth	Density: + Information: +	[86–88, 97]	
Electrostatic Hull	wraps superimposed shapes of the proteins sub-structure	Density: o Information: +	[17, 89, 90]	Physicochemical Properties
Spheres	incorporates structural variations as consequence of sequential rearrangements	Density: o Information: +	[91]	Physicochemical Properties
Distance Distribution	distribution of euclidean distances between each atom type	Density: o: Information: +	[92]	
Delaunay Triangulation	encodes the complete protein shape by finding the optimal edges between representative atoms	Density: o Information: +	[93, 94]	

+ (good), o (neutral/no declaration), − (bad) (see Table 1 for further details)

**Table 3:** Summary of alternative encodings (see Table 1 for further details)

Encoding	Description	Summary	Used in	Used along with
Chaos Game Representation (CGR)	a visual encoding of a sequence, generating a fractal	Density: - Information: o	[98–102]	Physicochemical Properties
Linguistic Model	description of AMPs by a grammar	Density: o Information: o	[103]	

### Sequence based encodings

#### Sparse encoding

The first approach that has been used to describe a peptide sequence is sparse encoding (also named binary encoding). In sparse encoding, each amino acid is represented as an one-hot vector of length 20, where each position, except one, is set to 0. Thus, in a vectorized format, the amino acids alanine and valine are encoded as 10000000000000000000 and 00000000000000000001, respectively [15]. For instance, the amino acid sequence GHKARVLAEAMSQVTGSAAVM, the p2 peptide ([16, 17]), is encoded into the matrix A as:

$$ A={\displaystyle \begin{array}{l}\\ {}\begin{array}{l}G\\ {}H\\ {}\vdots \\ {}V\\ {}M\end{array}\end{array}}\kern0.5em {\displaystyle \begin{array}{l}A\kern0.5em R\kern0.5em N\kern0.5em D\kern0.5em C\kern0.5em E\kern0.5em Q\kern0.5em G\kern0.5em H\kern0.5em I\kern0.5em L\kern0.5em K\kern0.5em M\kern0.5em F\kern0.5em P\kern0.5em S\kern0.5em T\kern0.5em W\kern0.5em Y\kern0.5em V\\ {}\left(\begin{array}{llllllllllllllllllll}0& 0& 0& 0& 0& 0& 0& 1& 0& 0& 0& 0& 0& 0& 0& 0& 0& 0& 0& 0\\ {}0& 0& 0& 0& 0& 0& 0& 0& 1& 0& 0& 0& 0& 0& 0& 0& 0& 0& 0& 0\\ {}\vdots & \vdots & \vdots & \vdots & \vdots & \vdots & \vdots & \vdots & \vdots & \vdots & \vdots & \vdots & \vdots & \vdots & \vdots & \vdots & \vdots & \vdots & \vdots & \vdots \\ {}0& 0& 0& 0& 0& 0& 0& 0& 0& 0& 0& 0& 0& 0& 0& 0& 0& 0& 0& 1\\ {}0& 0& 0& 0& 0& 0& 0& 0& 0& 0& 0& 0& 1& 0& 0& 0& 0& 0& 0& 0\end{array}\right)\end{array}} $$

Since machine learning models require a fixed input dimension, the respective sequence lengths have to be adjusted before encoding. In the present case, this happens either by a multiple sequence alignment or with a pairwise alignment against a reference sequence. The alignments will introduce gaps, hence a further dummy amino acid has to be added to the matrix. On the one hand, sparse encoding offers the advantage of providing an easy representation of the 20 proteinogenic amino acids (plus one dummy residue for gaps). On the other hand, the resulting input space for subsequent machine learning is inflated and could impose problems, such as the curse of dimensionality [18]. The feature vector dimension will be inflated to 21*max(l), whereby l denotes the length of a given peptide sequence. Nevertheless, sparse encoding is frequently used. For instance, Hirst et al. (1992) used this encoding to train a neural network and to predict secondary structure as well as the function [15]. However, the authors used sliding windows to separate the original sequence into segments such that the impact of spatially close residues is considered. Thus, the dimension of the input vector is 20 (each amino acid) times the window size [15]. Another study combined sparse encoding and a substitution-matrix-based encoding to predict peptide binding affinity to T-cell epitopes using neural networks [19]. The latter encoding increases the generalization ability of the classifier, whereas the sparse encoding does not provide additional information, except simply the amino acid itself [19]. This drawback of sparse encodings has been recognized by others. For instance, as part of a study to predict peptide induced modulation of antigen presenting cells, Nagpal et al. (2018) encoded the N-terminus and the C-terminus as binary vectors and used this encoding along with the overall amino acid composition as features for a support vector machine (SVM) [20]. Usmani et al. (2018) used a similar combination of sparse encoding of both termini and amino acid composition in order to predict antitubercular peptides by means of an ensemble classifier [21]. In addition, they state that sparse encoding has the advantage to keep the sequence order information [21].

#### Amino acid composition

**Fig. 1:**
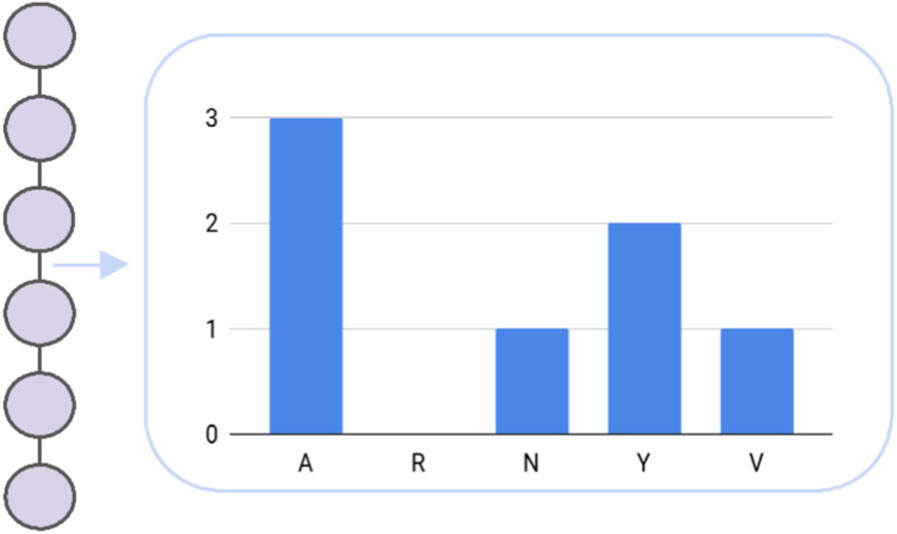
The single letter amino acid composition counts the occurrence of the respective amino acids

An approach to overcome the limitations of sparse encoding and hence making the resulting feature space more dense, is the representation of the amino acid sequence as its respective composition. Here, the final feature vector contains at each position the proportion of an amino acid in relation with the sequence length (Fig. 1). For instance, one can divide a peptide into chunks including both termini and calculate the local amino acid composition [22]. The amino acid composition differs from one class to the another and, for instance, cell penetrating peptides require hydrophobic residues at the N-terminus, which could be approximated well by the features gained from the local composition [1]. Additional performance has been achieved by introducing a technique called distance frequency, which calculates the distance between amino acids of similar properties and bins the occurrence according to the gap length. Matsudo et al. (2005) used both encodings to predict the subcellular location by means of SVMs [22]. Commonly, amino acid composition is applied to distinguish between different classes of peptides, i.e., antimicrobial and non-antimicrobial peptides [23] or to classify antiviral, antitumor, antibacterial, and antifungal peptides [24]. The former introduces quantitative matrices as a novel descriptor, which encodes the propensity of each amino acid at a certain position. This encoding has been employed in addition to local sparse encoding for analysing as well as predicting antimicrobial peptides in general. In contrast, the latter study applied increment of diversity (ID) to classify unknown peptides to the respective classes. To ensure a well-performing classifier, the ID is not only based on the amino acid composition, but is rather used along with the dipeptide and the pseudo-amino acid composition, which will be introduced hereinafter. Dubchak et al. (1995) proposed an encoding, which describes the composition (C), transition (T) and distribution (D) of similar, hence in terms of physicochemical properties, amino acids along the peptide sequence [25]. C refers to the composition of the respective residues, T denotes the frequency of the transition from one group to another and finally, D reflects the distribution of properties within 0, 25, 50, 75 and 100% of the sequence. The CTD-descriptor has been employed to predict protein folding classes [25].

#### Pseudo-amino acid composition

**Fig. 2:**
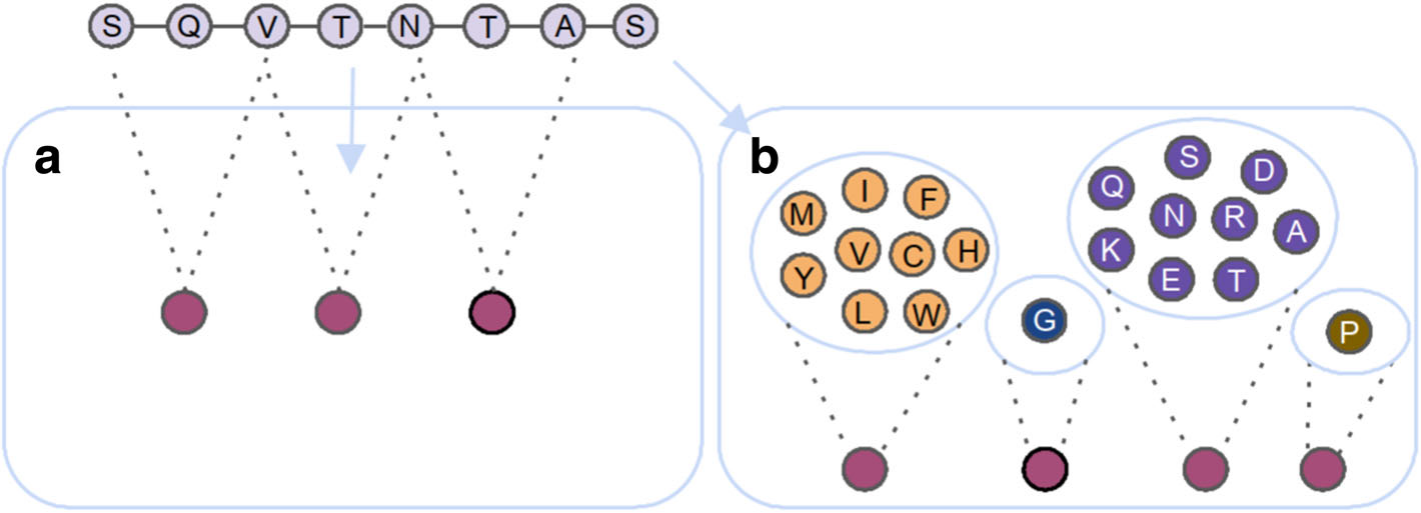
Sketch of sequence-based encodings derived from autocorrelation and reduced amino acid alphabet. **a** Autocorrelation and pseudo-amino acid composition from adjacent residues, considering a gap size of one. **b** Reduced amino acid alphabet. Clustering corresponds to similar physicochemical properties, according to Veltri et al. (2017)

Sparse encoding and the amino acid composition do not take into account the sequence order effect. This effect considers the vast amount of possible amino acid combinations as the sequence length increases. That is, for a peptide of length 6, there are already 20^6^ = 64,000,000 different sequence arrangements. In terms of antimicrobial activity, Cherkasov et al. (2009) pointed out that, albeit having very similar amino acid compositions, some peptides were virtually inactive [26]. Thus, the pseudo-amino acid composition (PseAAC) has been introduced to consider the effect of the sequence order [27]. The PseAAC computes the correlation between different ranges among a pair of amino acids, which leads to a 20 + λ dimensional vector (Fig. 2a). The first 20 components are the composition of the 20 natural occurring amino acids, whereas the 20 + 1 to 20 + λ components describe the correlation according to the respective sequence order level. For the most contiguous (λ = 1) and the second-most contiguous (λ = 2) amino acids, the PseAAC results in a 22-D (dimensional) vector. Thus, for λ = 1 the sequence order for adjacent amino acids are taken into account. The correlation function incorporates several physicochemical properties, such as the hydrophobicity and amino acid side chain mass. To verify that this method leads to a lower loss of information compared to the usual amino acid composition, several similarity measures have been employed. These include the prediction of subcellular locations of proteins, membrane protein types, as well as their particular locations [27]. To improve prediction accuracy, the PseAAC has been used by several studies, e.g., [28, 29], and [30], in combination with other types of encodings. For instance, in order to predict AMPs and additional efficiencies towards, e.g., cancer cells and HIV, PseAAC was applied in a two-level approach: first, it was used to encode peptide sequences to distinguish between AMPs and non-AMPs and second, to determine additional effects. Both classifications have been conducted by means of fuzzy k-nearest neighbors [28]. Moreover, additional physicochemical properties have been used to enhance the discriminative power of PseAAC [28]. Chen et al. (2016) tried to unveil novel anticancer peptides by enhancing the default dipeptide composition with PseAAC [29]. This approach considers long range interactions between amino acid pairs along with the dipeptide composition. The latter might reflect structural interactions, such as hydrogen bridge bonds between spatial close amino acids to form alpha helices [31]. An extension to the interaction of multiple encodings, including PseAAC, has been conducted by Meher et al. [30]. They used PseAAC in addition to structural and physicochemical encodings in order to distinguish between AMPs and non-AMPs. Again, an SVM was used to conduct the classification [30].

#### Reduced amino acid alphabet

**Fig. 3:**
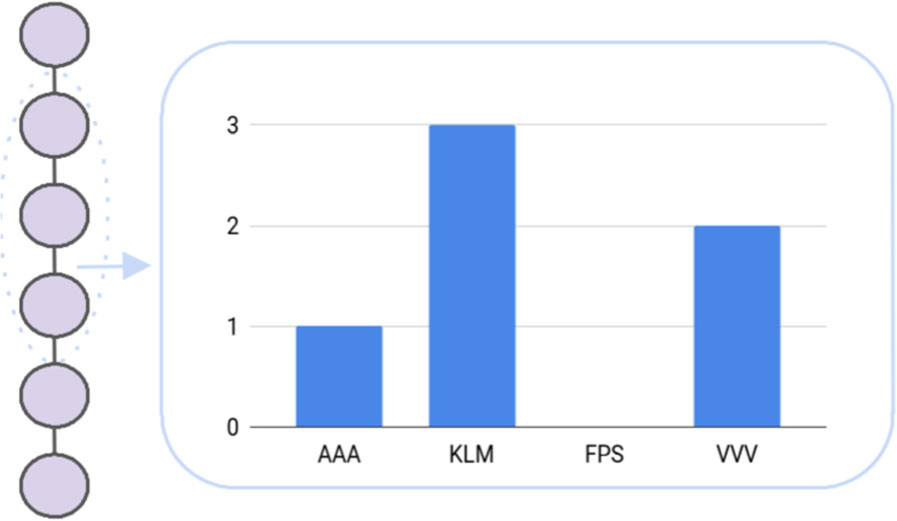
Similarly to the amino acid composition, the k-mer composition counts the presence of k-mers. In this example k is set to three

Sparse encoding, amino acid composition, and PseAAC consider, more or less, the actual amino acid sequence to encode a peptide. Therefore, the encoding might not reflect sequence variations well and this might negatively contribute to the classifier performance. In order to improve generalization, also considering mutations, one could make use of the reduced amino acid alphabet. Here, similar amino acids are grouped together, based on physicochemical, such as hydrophobicity and hydrophilicity [9] or structural properties, e.g., the backbone structure (Fig. 2b) [32]. The reduced amino acid alphabet has been employed in combination with the n-gram model to ease the classification of protein sequences. The n-gram model counts the occurrences of n-mers for an alphabet of size m, leading to a m^n^ dimensional, sparse representation of the initial sequence (Fig. 3). Nevertheless, despite the preceding alphabet reduction, the increased dimensionality is again a major drawback of the n-gram model. Thus, single value decomposition [33] has been applied to reduce the number of features to efficiently train an artificial neural network (ANN). Finally, the ANN is used to assign the query proteins to the respective protein families [9]. Comparable to the n-gram model, the n-peptide composition leads, in particular for an increasing n, to an inflation of the feature space. Yu et al. (2004) used the n-peptide model to predict the subcellular location of proteins in Gram-negative bacteria [34]. For this purpose, the dipeptide, amino acid, as well as the partitioned amino acid composition have been leveraged. For the latter, the sequence is split into equal-length segments and these segments are used to train several SVMs. The assignment of the respective subcellular location is then based on a majority vote of all classifiers [34]. Furthermore, the reduction of the amino acid alphabet, based on structural properties, has been used as the initial step to construct more complex features. These complex features consist of compositional, positional, position-shifted, and correlated features, which are combined through several boolean functions, such as matches and/or matchesAtPosition. The ultimate goal of the study was the prediction of AMPs and their selectivity for different kinds of bacteria and to this end, the complex features are further reduced by means of a filter-based feature selection [35, 36]. Another study uses the k-nearest neighbor clustering to aggregate amino acids into five classes using their amino acid index, i.e., amino acids with the respective highest (T), high (H), medium (M), low (L), and lowest (B) values of a particular physicochemical property are clustered together. This encoding (AAIndexLoc) is extended by the five-level dipeptide composition, which extends the aforementioned clustering by aggregating pairs of amino acids, such as TT, TH, and so forth. Along with these descriptors, Tantoso et al. (2008) employed the amino acid composition, for both termini and the middle part of the peptide, which leads to a dataset of 70 features for an SVM to predict subcellular location [37].

#### Physicochemical properties

**Fig. 4:**
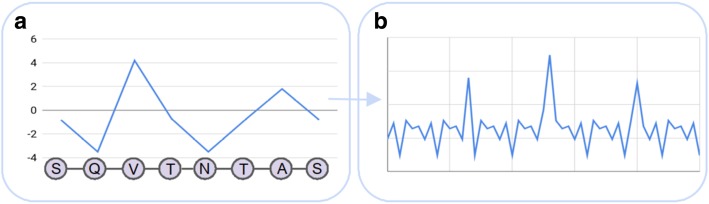
Sketch of sequence-based encodings derived from physicochemical properties and Fourier transformation. **a** The numerical representations are based on the physicochemical properties of Serine (S), Glutamine (Q), Valine (V), Threonine (T), Asparagine (N) and Alanine (A). **b** Fourier transformation derived from the encoded peptide sequence

One of the important encodings in AMP prediction, if not the most important one, is the translation of an amino acid to a particular physicochemical property, which have been determined in various wet lab experiments (Fig. 4a). The amino acid index database (AAindex) has been established as a unified source for these descriptors [38]. The AAindex is grouped into three parts, whereby the AAindex1 contains the just mentioned biochemical properties (one for each amino acid) and the AAindex2 aggregates different substitution matrices, such as the PAM250 or the BLOSUM62. The AAindex3 provides protein contact potentials, hence empiric values for spatial close amino acids, such as the Gibbs free energy change, to indicate preferred interactions between residue pairs [39]. The AAindex database, as a consistent source for numerical amino acids indices, has proven its usefulness in several studies. An example is the prediction of transmembrane protein segments [40]. Deber et al. (2001) used, among others, the hydrophobicity scale introduced by Kyte and Doolittle [41], as a reference to their experimental derived values of hydrophobicity [40]. The program annotates α-helical regions in the query sequence, based on the respective hydrophobicity and helix tendency thresholds [40]. So called z-descriptors have been employed as part of the prediction of cell-penetrating peptides [42]. These type of peptides reveal an important property, as they are capable to introduce macromolecules into the cell, which is especially interesting for the pharmaceutical industry [43]. The z-descriptors are derived from the principal components of physicochemical properties by means of partial least squares (PLS) projections [44]. PLS leads to a subset of five final features, capable to describe the 20 proteinogenic as well as 67 additional amino acids. The first three components can be considered as lipophilicity, volume (steric bulk), and polarity, respectively, whereas the fourth and the fifth component are not clearly derivable [44]. These properties are appropriate for the cell-penetrating peptide prediction, due to the intrinsic properties, which are the polarity (positively charged residues are advantageous) as well as the the amphi- and hydrophobicity [42]. However, Hansen et al. (2008) pointed out, that the method benefits from averaging z-descriptors, because that allows to compare sequences with varying length [42]. Nevertheless, to deal with varying protein or peptide sequence lengths, interpolation techniques have been introduced [45]. Sequence interpolation refers to a method, which connects multiple points, that is amino acid indices, via different linear and nonlinear functions. In order to obtain a continuous feature vector, the amino acid sequence is first mapped to the respective physicochemical property, followed by the actual smoothing, employing one of the interpolation functions [45, 46]. Physicochemical representations of peptides have been utilized to classify AMPs and non-AMPs [47]. To this end, Torrent et al. (2011) investigated the different characteristics of antimicrobial peptides, such as the isoelectric point, in-vivo aggregation, and hydrophobicity with respect to their discriminative power [47]. A peptide is described by its different characteristics and the particular averages were fed into an ANN to obtain the class to which the query peptide belongs [47]. In addition, the physicochemical property encoding is employed by various web servers for peptide retrieval, i.e., database queries, as well as for classification. Two examples are AVPpred [48] for antiviral peptide prediction and DBAASP for structure and activity of AMPs [49]. Moreover, this encoding has been used as part of several other studies to predict antimicrobial effects of synthetic peptides [50] or to find substructures with antimicrobial potency in larger proteins [51]. In order to take into account that some traits of AMPs are dependent on particular parts within the sequence, such as a positively charged N-terminus, further studies elucidated the physicochemical property dependence with respect to different sequence sections. One of these studies divided AMPs into datasets for both termini, calculated the physicochemical representation, and finally uses an SVM for classification on the best performing feature subset [52]. Another study leverages pattern changes of amino acid characteristics along a peptide sequence for the prediction of antimicrobial peptides by means of random forests (RF) [53]. An alternative approach, which leverages hydrophobicity values, is designated as the d-descriptor [54]. This encoding is founded on sequence moments, a two dimensional extension of sequence profiles. The amino acid sequence is squeezed between the y- (N-terminus) and the x-axis (C-terminus) with gradually bending of the single amino acids and subsequent vector summation. The length of the vectors arise from the respective property and the angle results from the amino acids orientation in the 2D space. Finally, the sequence moments are mapped to scalar values, which is named the d-descriptor. Juretić et al. (2009) used the latter in order to estimate the therapeutic index, the ratio of hemolytic and antimicrobial activity [54]. Finally, owing to the high dimensional feature vectors, if one uses all possible amino acid indices, several studies, such as [52], performed statistical analysis in order to reduce the features before the accomplishment of the actual experiments. Other studies used techniques such as PCA to obtain the aforementioned z-descriptors as well as factor analysis in order to describe all amino acids with only five factors [55]. Recently, Boone et al. (2018) proposed a classification method by means of the rough set theory [56]. To this end, physicochemical properties have been used to encode the samples and afterwards the algorithm finds suitable boundaries to differentiate between antimicrobial and non-active peptides [56].

#### Autocorrelation

An approach to consider physicochemical properties not only for a specific position, but also for amino acids which might be related in higher dimensional protein structure assemblies, can be described by an encoding, which is known as autocorrelation. In general, autocorrelation describes the interdependence between two distant signals in a time series, whereby the distance or the lag, respectively, is predetermined and fixed for a particular computation (Fig. 2a). For amino acid sequences, repeating patterns, i.e., a certain periodicity, might be unveiled [57]. In peptide, or generally in protein science, two algorithms to detect spatial autocorrelation have been employed: the Moron autocorrelation, which considers the local dependence of amino acids [58] as well as the Broto-Moreau autocorrelation, which describes the global relationship of the residues [59]. These formulas yield either positive values, meaning that amino acids with similar physicochemical properties follow each other (positive autocorrelation) or negative values, i.e., amino acids with different physicochemical properties are interconnected (negative autocorrelation). Values near zero point to no or less autocorrelation [60]. One of the earliest applications of autocorrelation was the statistical analysis of protein content [60] and the prediction of α-helices [57]. A noteworthy relationship exists between autocorrelation and PseAAC, since both take the sequence order effect into account, by measuring the correlation among amino acid pairs. Further advantages of this encoding are the reduction of the feature space as well as the normalization of the sequence length [61]. To this end, this descriptor has been utilized in several studies and facilitated, for instance, the prediction of mutation induced stability alterations of the gene V protein by bayesian-regularized genetic neural networks [61]. Another study dealt with protein-protein interactions and used the autocorrelation descriptor to train the rotation forest algorithm [58]. Furthermore, Kleandrova et al. (2016) used this encoding for the prediction of antimicrobial activity in known peptides as well as for screening of novel, artificial AMPs [59].

#### Substitution and scoring matrix

Substitution matrices, such as BLOSUM62 or PAM250, represent accepted mutations between amino acid pairs, i.e., sequence alterations with either no or positive impact in terms of the protein function. More specifically, it is the likelihood for a specific mutation within a certain time frame [62]. In contrast, the position-specific scoring matrix (PSSM) describes, based on a initial BLAST alignment, and iterative refinement, how amino acids are evolutionary conserved at a specific position. This results in positive values for a highly conserved residue and negative values for the others. Values near zero indicate weakly conserved residues [63]. Alignments with PSSMs can be regarded as an extension of substitution matrices, since instead of using, e.g., the PAM250, the PSSM is used for the alignment score, which leads to improved substitution probabilities and hence more sensitive alignments [64]. With regard to antimicrobial peptides, this encoding weights functional important residues stronger, such that conclusions for antimicrobial effects can be drawn and hereof facilitates querying peptides with unknown activity. For instance, the BLOMAP-encoding incorporates the BLOSUM62 to calculate distances in a high dimensional input space, i.e., the substitution matrix, to a lower dimension, using the Shannon-projection [65]. Maetschke et al. (2005) demonstrated how this descriptor improves signal peptide cleavage site prediction using, among others, Naïve Bayes (NB) and ANNs [65]. Due to the ambiguity of some BLOSUM50 entries, i.e., same values for amino acids, which in fact differ towards their physicochemical properties, Huang et al. (2005) utilized this substitution matrix in order to extend the sparse encoding [66]. They replaced each non-zero value with the respective BLOSUM50 score, such that the information of a particular amino acid is kept and additional information, derived from the substitution probabilities, is taken into account. The adjusted encoding has been used to predict T-cell epitopes by means of an SVM [66]. Karypis et al. (2006) applied substitution matrices to train SVMs for protein secondary structure prediction [67]. Therefore, k-mers are generated and mapped by means of the PSSM and BLOSUM62 matrices, respectively, to their numerical encoding. A binary SVM has been trained on this input and the results of this classification are used along with the aforementioned encoding for a second classification, which incorporates both [67]. Kumar et al. (2008) employed PSSMs as the encoding for a SVM to predict RNA binding sites in proteins [68]. Another study builds several SVMs using different encoding schemes, such as split-, dipeptide-, and regular amino acid composition together with PSSMs to enable the prediction of malaria parasite mitochondrial proteins [69]. Furthermore, the classification of bacterial virulent proteins has been facilitated through the usage of sequence order effect conserving descriptors like PseAAC, the PSSM, and the above mentioned AAIndexLoc encoding. Nanni et al. (2012) used SVMs as well as an ensemble classifier approach for the final protein identification [70]. The latter employs a two-stage feature transformation method, which couples PCA and neighborhood preserving embedding, followed by decision trees [70]. In order to reveal DNA-binding proteins, Xu et al. (2015) extended PSSMs to incorporate dipeptide composition, which allows the computation of the probability of simultaneously appearing pairs of same and different amino acids within a certain distance along the peptide sequence [71].

#### Fourier transformation

Fourier Transformation (FT) can be used to detect underlying patterns in time series by transforming the time signal to a frequency domain (Fig. 4b) [72]. Examples for the application in biomedicine are the detection of the repeated occurring of coding and non-coding regions in DNA sequences and the prediction of cellular locations of proteins [73]. FT has been applied as part of a study to discover peptides with antimicrobial activity [73]. To this end, the residues have been first mapped to physicochemical properties, followed by the actual FT. Afterwards, the similarity between a reference peptide and potential hits has been measured by means of the Euclidean distance between the respective power spectra [73]. Moreover, Yin et al. (2017) proposed an approach to predict protein-protein interactions by means of discrete Fourier transformation (DFT) [74]. They showed, that the detection of coevolution patterns can be carried out without using multiple sequence alignments. Again, hydrophobicity values have been used to encode the amino acid sequences. Afterwards, subsequences have been extracted with a sliding window approach and transformed via DFT. Based on the DFT results, the evolutionary distances between proteins were calculated using the Euclidean metric. Finally, a protein-protein interaction is indicated by means of the Pearson correlation coefficient [74].

### Structure based encodings

The secondary structure of a protein or peptide, respectively, is mainly determined by its primary structure, i.e., the order of the amino acids [75]. Moreover, the peptide structure has a strong correlation with antimicrobial activity [76]. Thus, for the prediction of antimicrobial activity, it is reasonable to use sequence-based encodings, but, since the secondary structure cannot be completely derived from the primary structure, it is also conclusive to develop structure-based encodings. In addition, the employing of both descriptors simultaneously, allows the classifier a better generalization and thus improves the overall accuracy [77]. The following section introduces several applications of structure-based encodings.

#### Quantitative structure-activity relationship

An alternative approach to describe amino acids sequences by their chemical properties has been developed as part of quantitative structure-activity relationship **(**QSAR) studies. In essence, QSAR refers to the prediction of a particular property or activity by means of its molecular characteristics and structure [78]. This is also the crucial difference between the description of amino acids by their physicochemical properties and QSAR. The latter focuses solely on molecules, whereas the former encodes the whole residue. In addition, QSAR is mainly applied in chemoinformatics for high-throughput screening, i.e., to find novel active substances in databases using two- and three-dimensional representations of compounds [79]. However, several studies propose QSAR modeling based approaches to predict antimicrobial activity. For instance, one study uses this encoding^1^ to imitate the artificial AMP Novispirin G_10_ by similar peptides in order to enhance its potency. Here, molecular modeling was used to calculate 3D structure conformations. The structure was then used to obtain a set of descriptors, such as hydrophobicity, amphipathicity, and electrostatic charges. Finally, a subset of meaningful features have been determined and the activity measurement of the analogs was determined by predicting the amount of inhibited bacterial growth [80]. Moreover, Bhonsle et al. (2007) aimed to find informative 3D physicochemical descriptors in order to predict bioactivity of AMPs [81]. Solvent-accessible surface describing (e.g., fractional charged partial surface area), structural (H-bond acceptor) and spatial (density) descriptors, among others, turned out to be good indicators for antimicrobial activity [81]. Jenssen et al. (2007) investigated, whether there is a set of molecular descriptors, which can be used to optimize antimicrobial activity against *P. aeruginosa* [82]*.* This set encompasses the aforementioned z-descriptors as well as the contact energy between amino acids, inductive and conventional QSAR descriptors [82]. Similar descriptors have been evaluated in order to design AMPs in silico [83]. Shu et al. (2013) uses PCA to extract the first six principal components from topological and structural characteristics to predict antimicrobial activity of synthetic cationic polypeptides [84]. In contrast, Schneider et al. (2017) utilized molecular descriptors to train self-organizing maps (SOM) [85]. Afterwards, the continuous SOM responses are adjusted by means of lateral inhibition and utilized as input for a deep learning model in order to predict helical AMPs [85].

#### General structural encodings

**Fig. 5:**
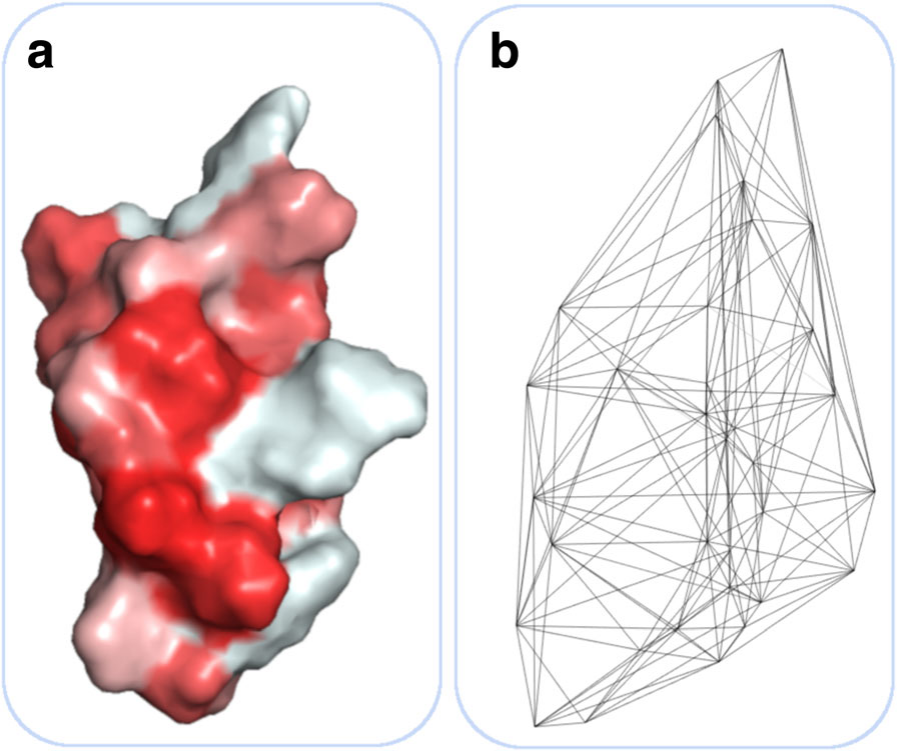
Exemplary structure-based encodings for antimicrobial peptide Human Defensin 5 (PDB:2LXZ). **a** Solvent accessible surface. Color coding according to hydrophobicity scale (Eisenberg et al., 1984) **b** Delaunay triangulation of the same peptide calculated from Cα-atoms. Bose et al. (2011) used the summed distances between amino acid pairs to encode protein structure

Unlike QSAR-based methods, general structural encodings map structure information derived from the whole peptide, to a numerical representation. The peptide structure is described by means of their total 3D shape. This is contrary to QSAR, because instead encoding an amino acid sequence from a molecular viewpoint, the whole peptide structure is considered (Fig. 5a). For instance, Cui et al. (2008) predicted the secretion of proteins into the bloodstream [86]. They used features including physicochemical properties as well as structural information, such as secondary structure, and solvent accessibility. The final prediction has been facilitated by an SVM [86]. Chang et al. (2015) employed conditional random fields (CRF) for probability prediction of critical regions along an AMP sequence [87]. CRFs are an algorithm similar to hidden Markov models, but more variables, such as the surrounding context, can be incorporated. In the present case, several structural descriptors along with physicochemical properties have been used for the prediction. The structure-based encodings encompasses the assignment of predicted secondary structure, conserved protein domains, predicted antimicrobial regions [88] as well as the aggregation tendency [87]. Dybowski et al. (2010) proposed a stacked classifier model to predict the HIV-1 tropism [89]. To this end, the authors trained two independent RFs, whereby the first used hydrophobicity values and the second used the hulls of the electrostatic potentials of the V3 loop, a short peptidic sequence of the viral gp120 protein, as descriptors. The electrostatic hull has been determined in order to acknowledge even subtle differences between different co-receptor tropisms as well as to wrap superimposed shapes of the peptides sub-structure. A third RF combined the output of the other models for the final class assignment [89]. Due to high computational effort during the calculation of the electrostatic potential, Heider et al. (2014) presented an extension of this method [90]. The authors leveraged, that the current model achieves good performance with a constant dielectric value and ionic strength, thus simplifying the calculation of the potential to Coulomb’s law. Finally, the electrostatic potential has been calculated based on the cluster centers. The centroids are determined by all points within a certain distance to the C_α_-atoms of the V3 loop [90]. As part of another study, the authors increased the prediction power by means of multiple RFs, combined to an ensemble classifier. The respective classifiers used physicochemical as well as structural properties to predict resistance against a novel HIV-1 maturation inhibitor.

The structural encoding is based on the aforementioned electrostatic potential. In addition, a genetic algorithm has been implemented to find an optimal subset of the physicochemical properties [17]. However, Bozek et al. (2013) pointed out, that the structural encoding of the V3 loop exhibits limitations, since only two physicochemical properties has been used for description [91]. To this end, they proposed a novel encoding, which incorporates structural variations as consequence of sequential rearrangements. Thus, based on the template structure, spheres, whose centers are depicted by reference atoms, are used to enclose spatial related residues of different loop variants. Afterwards, the averaged physicochemical properties of all residues within these regions are used to determine HIV-1 co-receptor usage [91]. In contrast, Sander et al. (2007) introduced an distance distribution approach in order to improve co-receptor tropism based on V3 loops [92]. This method calculates the euclidean distances between each atom type. Afterwards, the respective distances are used to obtain the underlying distribution. Finally, the feature vector is obtained by sampling from this distribution, leading to a final size of each possible combination times samples [92]. Nevertheless, HIV-1 is a very complex organism and hence, several strategies have been tackled in order to combat the virus, such as the aforementioned relation between the V3 loop and tropism as well as between mutations, structure and drug resistance [93]. To this end, another encoding has been developed to describe protein structure based on Delaunay triangulation (Fig. 5b). In essence, the Delaunay triangulation states that, if three points or vertices, respectively, are connected via edges, no further vertex must be located within the circumcircle of these three vertices. This encoding facilitates to encode the complete protein shape by finding the optimal edges between representative points, such as C_α_-atoms. Thus, it is able to incorporate information about spatial close residues, which might be lost by a descriptor based on the primary structure only. Finally, the feature vector consists of 210 entries, derived from the adjacency matrix of all amino acid pairs. The respective values are resulting from the averaged distance among these pairs [94]. Albeit this encoding has been mainly employed in the context of computational HIV research, it might work as well for antimicrobial peptides, owing to very good classification results of several studies [95]. To sum up, structural encodings are an appropriate extension to sequence-based encodings since antimicrobial activity is determined by the three-dimensional composition of the residues [96] and in addition, the combination of sequence- and structure-based encodings increases discriminating power [97].

### Alternative encodings

There are further encodings, which do not really fit into the proposed categories, i.e., sequence or structural encodings. One of these encodings, which are summarized in Table 3, is the Chaos Game Representation (CGR). In general, the CGR is a visual encoding of a sequence, generating a fractal. The sequence can be obtained, e.g., from random numbers or from biological sequences, such as bases (DNA) and amino acids (proteins). In the case of the former, numbers from 1 to 3 denoting a vertex of a triangle. The algorithm works as follows: firstly, a starting point *s* is determined and afterwards, one of the numbers is randomly selected as the target vertex *t*. The next point is located on the half way between *s* and *t*. By repeating this procedure, the so called Sierpinski triangle will be generated. The Sierpinski triangle is special about its recursively defined sub-structures, which are also triangles [98]. In the case of the DNA, *t* is not selected by chance, but rather by the successive base. Here, adenine (A), thymine (T), guanine (G) and cytosine (C) are the labels of a square. After conducting the algorithm, the resulting fractal shows lower order, but still exhibits notably patterns, originated from the underlying sequence. Moreover, points which are close in the CGR do not have to be necessarily adjacent in the sequence, which means that the CGR might introduce novel distance metrics of subsequences [98]. However, with respect to AMPs, CGR has been applied as part of a variety of studies in order to deal with amino acid sequences. As such, Basu et al. (1997) classified similar amino acids to 12 different groups, each representing a target vertex for the CGR algorithm [99]. In addition, the resulting dodecagon has been divided in 24 grids and the amount of points per grid has been used to predict the affiliation to protein families [99]. A further study reduced the amount of vertices to 8, whereby the grouping happened according to the respective physicochemical properties [100]. Moreover, He et al. (2016) extended the illustration to three dimensions, which results in a cube, rather than a planar octagon [100]. The study investigated how this encoding could be employed for multiple sequence alignments. To this end, the authors introduced a method, which computes the euclidean distance between amino acid pairs of two encoded proteins. Finally, the similarity of two proteins is denoted by the sum of the distances [100]. Recently, one study used CGR in a 10D space, using a hypercube for the prediction of anticancer peptides [101] as well as for protein-protein interactions [102].

Another method, which does not fit into the proposed sections has been introduced by Loose et al. (2006) in order to design novel AMPs [103]. In this study, the authors considered AMPs as a corpus of sentences and the goal was to examine, whether antimicrobial activity is described by a certain grammar. To this end, a linguistic model has been derived from active peptides and successfully employed for the design of AMPs [103].

## Models

So far, state of the art encodings have been discussed extensively. The next section will summarize the utilized learning algorithms. Popular models in antimicrobial peptide prediction include decision trees [21, 50, 71] and random forests [17, 53, 104, 105], but also neural networks have been employed in several studies [9, 26, 106]. Moreover, deep learning, as an extension to ordinary neural networks, has been applied frequently and thus a more detailed description, along with a summary in Table 4, is provided in the next section. Support vector machines are a further outstanding model in AMP prediction and were part of several studies [29, 30, 91]. In fact, there are specific kernels designed for amino acid based proteins/peptides sequences, known as string kernels. To shed some light into this topic, the upcoming section will highlight these kernels in more detail. In addition, Table 5 summarizes the presented kernels. However, besides the popular algorithms mentioned above, further methods leveraged partial least squares [82, 83, 107], hidden Markov models [108], logistic regression [109] and Bayesian networks [110]. Furthermore, ensembles of several classifiers have been also successfully implemented, such as in [17] or [21], whereby often one classifier is trained with a particular sequence or structural encoding. As part of an optimized feature set construction, genetic algorithms have been employed, by, e.g., Kernytsky et al. (2009) [111] as well as Veltri et al. (2017) [36]. Moreover, Krause et al. (2018) made use of genetic algorithms to optimize cell-penetrating peptides [43].

**Table 4:** Different encodings from deep learning models (see Table 1 for details)

Encoding	Description	Summary	Used in	Used along with
ProtVec	amino acid sequences are encoded as a distributed representation of k-mers	Density: + Information: +	[124]	
Voxel	structures of proteins are encoded as voxels	Density: o Information: +	[125, 126]	
Matrix	mimicks images by regarding the respective entries of PSSMs as pixel densities	Density: o Information: +	[127, 129, 130]	PSSM
Autoencoder	extracts representative characteristics in order to reproduce the input as good as possible	Density: + Information: o	[131]

**Table 5:** Different types of string kernels (see Table 1 for further details)

Encoding	Description	Summary	Used in	Used along with
Spectrum Kernel	generates all possible subsequences of length k and counts the occurrences of these k-mers	Density: - Information: -	[112]	
Mismatch Kernel	considers a certain distance, hence mismatches, between two k-mers	Density: - Information: o	[114–116]	General Structure
Distant Segment Kernel	allows a gap between two k-mers	Density: - Information: o	[118]	
Local Alignment Kernel	obtained from local alignment scores	Density: + Information: o	[119]	Spectrum Kernel, Mismatch Subsequence Kernel
Subsequence Kernel	measures sequence similarity, gaps within k-mers are taken into account	Density: + Information: o	[119]	Frequency of Amino Acid Pairs
Frequency of Amino Acid Pairs	similar to dipeptide composition	Density: - Information: o	[119]	
String Kernels + Physicochemical Properties	optimization of existing string kernels such that these involve physicochemical properties	Density: + Information: +	[120]	Physicochemical Properties
Generic String Kernel	string kernel with physicochemical properties and penalization of non adjacent segments	Density: + Information: +	[121, 122]	

### String kernel

Support vector machines (SVM) are capable to efficiently distinguish between binary input data by projecting the data to a higher input space, using kernel techniques [112]. Moreover, these kernel techniques allow a linear separation of a nonlinear classification problem, which is also known as the kernel trick [113]. One type of these kernels are string kernels, which are employed to measure sequence similarity [112]. In essence, the idea of string kernels implies that strings are mapped to a numerical representation in order to be used as input for an SVM. Thus, it is basically another encoding of an amino acid sequence, i.e., a method to map the string representation of peptide sequences to high dimensional feature vectors. Hence, several studies proposed corresponding methods, such as Leslie et al. (2002), who extended the spectrum kernel, in order to incorporate sequence variations, to the mismatch kernel [112]. The former generates all possible subsequences of length k and counts the occurrences of these k-mers within the query sequences, leading to a similarity metric based on shared k-mers [112]. This encoding is similar to the k-peptide composition, for instance the dipeptide composition (k = 2), which has been introduced earlier. The mismatch kernel on the other hand, considers a certain distance, hence mismatches, between two k-mers and takes into account, that similar sequences might have similar properties. Owing to the nature of spectrum kernels, further investigations revealed important and meaningful motifs. As a case study, the authors predicted homolog proteins [114]. Furthermore, string kernels have been applied to predict tumor suppressors, among others. Here, small molecules are encoded in their 1D, 2D, and 3D representations. In 1D, mismatch kernels have been employed to measure the similarity between the atomic sequences [115]. Another study investigated the performance of combined as well as weighted mismatch and structure derived similarity score kernels [116]. For these kernels, each entry in the feature vector is obtained from structure alignments between the input peptide and a peptide database [117]. The encoding incorporates the similarity to further peptides, whereby conserved peptides are depicted with higher scores. Boisvert et al. (2008) proposed an extension of the string kernel, which allows a gap between two k-mers [118]. Thus, the distant segment kernel takes into account the co-occurrence of remote sequence segments. The authors used this kernel in order to predict HIV-1 co-receptor tropism and achieved higher levels of accuracy compared to other methods [118]. Moreover, several string kernels have been employed and compared to predict linear B-cell epitopes [119]. These include the already introduced spectrum and mismatch kernel as well as the local alignment kernel, obtained from local alignment scores, and the subsequence kernel, which measures sequence similarity, similar to the mismatch kernel, albeit gaps within k-mers are taken into account. A third kernel measures the frequency of amino acid pairs (see dipeptide composition), which is due to a bias towards certain dipeptides in B-cell epitopes [119]. Toussaint et al. (2010) recognized that dealing with the sequence only might result in a loss of information [120]. For this reason, the aim of their study was the optimization of existing string kernels such that these involve physicochemical properties [120]. This kernel has been used by another study in conjunction with the penalization of non-adjacent segments, which finally has led to the generic string kernel for small molecules [121]. The authors applied this kernel in a subsequent study in order to detect antimicrobial peptides. All possible peptides with a specific length have been generated by means of source-to-sink graphs. In these graphs, all vertices are k-mers and all edges are weighted according to the antimicrobial activity, computed by means of the generic spectrum kernel. Finally, the detection of the most active peptide corresponds to the detection of the longest path within the graph [122].

### Deep learning

Machine learning algorithms based on artificial neural networks, especially deep learning models, have the advantage of incorporating automated encoding, i.e., feature generation. In general, the encoding results from several, successive connected layers, which work as filters for particular parts of the input [5]. However, these models require a large number of training examples in order to generalize well. Fortunately, owing to advances in next-generation sequencing technologies, biological sequences, such as peptides and proteins, are publicly available in vast amounts [123]. Several studies made use of that and showed how deep neural networks perform well on biological problems. For instance, Asgari et al. (2015) proposed a method called protein-vectors, which splits a sequence into k-mers to learn the context of these word representations [124]. Here, amino acid sequences are encoded as a distributed representation of k-mers, which were employed for protein family classification or the prediction of disordered proteins. This approach is derived from natural language processing and uses the context, hence the adjacent residues, for the central k-mers (“words”) syntactic and semantic description. The realization is carried out through building a sufficient large training corpus of protein sequences (“sentences”) by breaking all available sequences into overlapping k-mers. Afterwards, neural networks are used to find optimal, numerical representations, i.e., feature vectors, of the input sequences by means of the skip-gram model. By using these vectors, the authors showed that this framework encodes physicochemical properties well and high levels of accuracy have been achieved in the family classification task [124]. Jiménez et al. (2017) utilized deep learning to predict protein-binding sites [125]. To this end, the structures of proteins are encoded as three-dimensional objects, whereby a cubic segmentation in so-called voxels, which are 3D pixels, takes place beforehand. The encoding of each of these cubes is based on the contained atoms. In order to incorporate physicochemical properties, the input is further upscaled to 8 property channels [125]. A similar approach has been elaborated by Amidi et al. (2018) to predict enzyme classes [126]. Again, protein structures are encoded as voxels and are used as input for a convolutional neural network (CNN), but in contrast to Jiménez et al., the orientation of the protein has been considered. The authors point out, that the structure orientation in the Protein Data Bank (PDB) does not capture the dynamic of the protein and consequently used the proteins barycenter as origin and the first principal components for the orientation of the coordinate system. Overall, the model achieves good accuracy [126]. Another study uses position-specific scoring matrices (PSSM) as 2D input for CNNs, hence mimicking images by regarding the respective substitution probabilities as pixel densities. The studies goal is the automated partitioning of efflux proteins families [127]. This class of proteins provide an important tool for multi-resistant pathogens, because they allow them to convey molecules out of the cell, thus lowering the overall concentration of antibiotics [128]. Two further publications deal with alignment-free comparison of sequences, using CNNs. Both methods encode the input sequences as two-dimensional one-hot matrices, leveraging the convolutional layers for unveiling of latent features. Seo et al. (2018) employed this approach in order to predict protein families [129]. However, Zheng et al. (2018) extended this approach by training of two identical neural networks (siamese neural networks), which allows to compare sequences with respect to their dissimilarity [130]. These two methods, as well as the earlier introduced ProtVec [124], have in common that they aggregate amino acid sequences of varying lengths to a fixed-length numeric vector of lower dimension. Since this feature reduction keeps intrinsic properties of the proteins, these algorithms might serve as potential encodings for AMPs. Similar to this CNN based dimension reduction are autoencoders. Autoencoders are applied to learn a dense representation of the input, i.e., to extract representative characteristics in order to reproduce the input as good as possible. For instance, Wang et al. (2017) employed stacked autoencoders to predict protein-protein interactions [131].

## Databases and packages

Having access to existing data sets is crucial to push computational, antimicrobial peptide prediction further. Thus, several projects aim to enable researchers a public database to active peptides. Consequently, this part introduces established databases and highlights some characteristics of these web services. Although data access is granted, there are still a plenty of possible encodings for testing. Fortunately, there are ready-to-use implementations of many encodings and the subsequent section lists a choice of these handy packages.

### Databases

Piotto et al. (2012) presented YADAMP (yet another database of antimicrobial peptides) [132]. The authors collected the data sets, i.e., AMPs, from various, published studies. Potential hits can be limited, e.g., by specifying certain physicochemical properties and/or target organisms. Respective results provide more details with respect to activity and structural properties [132]. CAMP (collection of antimicrobial peptides) obtains AMP sequences and structures from well-known protein databases, such as UniProtKB [133]. Active peptides have been filtered out via keyword search. By providing several links to further web services, CAMP is a comprehensive resource for AMPs as well as active peptides in general [133, 134]. Wang et al. (2016) published the third update for the antimicrobial peptide database (APD3) [135]. Besides its focus on natural occurring AMPs, this database stores various active peptides, e.g., anti-HIV, spermicidal, and for wound healing. A web form lets the user specify custom query parameters, such as physicochemical properties [135]. Pirtskhalava et al. (2016) extended the database of antimicrobial activity and structure of peptides to the second version (DBAASPv.2) [49]. The service provides, among further details, potency values against several pathogens, described by inhibition coefficients. Moreover, the authors conducted molecular modeling for unveiling unknown structures of AMPs [49]. Finally, a comprehensive data repository of antimicrobial peptides (DRAMP) has been set up by Fan et al. (2016) [136]. They included additional features, hence similarity search, sequence alignment, and conserved domain search, besides established tools, which already have been introduced by other [136]. More information about web services for AMP retrieval can be found in two recent studies, published by Porto et al. [137] and Gabere et al. [138].

### Packages

As mentioned before, many of the sequence-based encodings have been implemented in user-friendly packages, using, e.g., R^2^ or Python.^3^ Interpol is an R-package for normalizing peptide sequences to a uniform length, using different interpolation methods and descriptors of the AAindex database [45]. Cao et al. (2013) developed propy, which provides Python access to methods for amino acid composition, autocorrelation and pseudo-amino acid composition (PseAAC), among others [139]. In contrast, protr, implemented by Xiao et al. (2015), provides similar methods for the R programming language [140]. In addition, all methods can be accessed through a public web server. However, the web interface lacks the possibility of passing custom method parameters and is hence only recommended for ad-hoc calculations [140]. Ofer et al. (2015) released ProFET, i.e., protein feature engineering toolkit, a Python-based distribution with a variety of ready-to-use amino acid encodings [141]. Among default encodings, which have been implemented by others, this package offers also reduced amino acid alphabet, autocorrelation, amino acid propensities, as well as transformed CTD features [141]. modlAMP is a Python library specifically developed for antimicrobial peptides. Besides a selective choice of encodings, Müller et al. (2017) added methods for the whole prediction pipeline, i.e., sequence retrieval, visualization, and machine learning algorithms [142]. Moreover, performant model parameters can be obtained automatically via a grid search [142]. In contrast, POSSUM (position-specific scoring matrix-based feature generator for machine learning) is a toolkit, which facilitates the representation of amino acids with PSSM derived encodings [143]. Wang et al. (2017) published POSSUM as a public web server as well as a Perl/Python-based tool, executable via the command line [143]. PyBioMed is another Python library foremost aiming cheminformaticians, owing to the fact, that many molecular encodings are implemented, e.g., topological descriptors, applicable in QSAR studies [144]. Nevertheless, Dong et al. (2018) rounded out this package with a variety of amino acid encodings and additional tools, such as sequence and structure retrieval [144]. Recently, Chen et al. (2018) published iFeature, which is accessible as a Python package and web server [145]. This tool adds functionality in order to encode amino acids based on AAindex entries as well as structure-based encodings, such as accessible surface area and main-chain-torsional angles. Moreover, algorithms for clustering, feature selection, and dimensionality reduction are available [145].

## Encoding selection

It is quite challenging to find a suitable encoding within the variety of possibilities, thus, this section provides recommendations for the selection process. This might be helpful for computational biologists, due to the fact, that, as far as we know, no guidance of an appropriate encoding selection has been published until now. Unfortunately, it is not easy to provide generally applicable processes, which encoding will work for a particular application, thus we follow the approach from Heider et al. (2014) [90] and propose the measurement of diversity as a rule of thumb [146], until more sophisticated techniques have been unveiled. In order to calculate the diversity, it is necessary to train various classifiers on different encoded peptide data sets and combine the outputs. In particular, the diversity is based on the decision of single classifiers with their respective strengths and weaknesses. Thus, we suggest to conduct the encoding selection in such a way, that the ensemble maximizes the disagreement measure D, which is the probability of the disagreement between the classifier i and j, which minimizes the correlation of two classifiers i and j, as well as maintains the overall prediction accuracy [90]. The disagreement measure D is defined as:

$$ {D}_{i,j}=\frac{1}{n}\ast \sum \limits_n^{k=1}\mid {o}_k^i-{o}_k^j\mid $$

Here, o^i^ and o^j^ refer to the outputs of classifier i and j. Furthermore, we recommend to combine sequence and structure based encodings. For more details we refer to [90]. A comprehensive introduction into the diversity of classifier ensembles can be found in [146].

## Conclusions

The amount of effort that has been expended in the last decades, demonstrates how important and essential efficient encodings are for detection of peptides with antimicrobial activity. This is reflected by diverse approaches and methods, which have been proposed in numerous publications. In the current study, we tried to aggregate existing, useful encodings and models, specifically for antimicrobial peptide (AMP) classification for multi-resistant pathogens. But also as part of other protein or peptide studies, respectively, promising encodings have been developed. In particular, sequence- and structure-based encodings have been discussed along with their applications. As part of sequence representations, major encoding schemes as well as different customizations are introduced. Moreover, structural encodings encompassed molecular as well as general representations and a particular focus was set again on application dependent customizations. Finally, a selection of alternative encodings, beyond sequence- and structure-based encodings, are presented. The second part highlighted employed models as well as string kernels as encodings for support vector machines. Deep learning is a popular machine learning method and requires little or no encoding for the classification process. Nevertheless, exciting applications in protein research can be found in literature and thus, have been covered as well. As mentioned at the beginning, this review summarized encodings specifically for AMPs, however, every machine learning based protein/peptide classification task can be tackled by means of the proposed techniques. Moreover, to enhance research capabilities, several studies already implemented many of the reviewed encodings and published ready-to-use packages in commonly used programming languages. Again, this review collected most popular ones and provides an unified source of these. In order to lower obstacles further, we added a separate section about existing antimicrobial sequence databases. In conclusion, this review provides a common basis of methodologies in theory as well as practical tools to promote AMP research. Due to the fact, that we emphasized on encodings derived from AMP classification tasks, it is not surprising, that a large number of further techniques for amino acid representation exist, which, for obvious reasons, could not covered in this review. Moreover, additional research is required in order to incorporate the structure of AMPs and to examine whether the simultaneous encoding of sequence and structure can increase the prediction performance further. Nevertheless, many studies showed already at this point very good results. The engineering of amino acid encodings supports not only the detection of novel AMPs and consequently the battle against multi-resistant pathogens, but could also impact other major diseases, such as HIV and cancer. Research must be continued in each direction, in order to leverage the full potential of AMPs. To this end, besides the aforementioned simultaneous deployment of sequence- and structure based encodings, we propose further approaches. Delaunay triangulation is a promising encoding for peptide structure. By integrating additional information, e.g., physicochemical properties, to the graph, one could leverage advantages of both. In order to ease the access, this, as well as structure encodings in general, might be provided in a separate library. Moreover, since implementations exist for R and Python and each language provides a unique set of encodings, it is beneficial to develop a package, which provides those, that are not covered by an existing one. Finally, a comparative study is necessary to examine the potential of single encodings on a range of independent, biomedical data sets. Thus, encodings could be revealed, which are preferable for a designated application.

## Abbreviations

AMP: Antimicrobial peptide
ANN: Artificial neural network
CGR: Chaos game representation
CNN: Convolutional neural network
CRF: Conditional random fields
DFT: Discrete fourier transformation
FT: Fourier transformation
ID: Increment of diversity
NB: Naïve Bayes
PDB: Protein data bank
PLS: Partial least squares
PseAAC: Pseudo-amino acid composition
PSSM: Position-specific scoring matrix
QSAR: Quantitative structure-activity relationship
RF: Random forest
SOM: Self-organizing maps
SVM: Support vector machine

## Footnotes

1 Since QSAR actually refers to the general model, the abbreviation will be used from now on interchangeable with the molecule property encodings.

2
https://www.r-project.org/

3
https://www.python.org/
